# CareVR, a Virtual Reality assisted communication training programme to support informal carers of people with psychosis: a feasibility study

**DOI:** 10.1186/s12888-026-08203-w

**Published:** 2026-06-01

**Authors:** Juliana Onwumere, Laurence Rogers, Kalya Win Aung, Jerome Di Pietro, Lucia Valmaggia

**Affiliations:** 1 Department of Psychology, Institute of Psychiatry, Psychology & Neuroscience, King’s College London, 16 De Crespigny Park, Denmark Hill , London, SE5 8AF UK; 2 Bethlem Royal Hospital, South London and Maudsley NHS Foundation Trust, Beckenham, London, UK; 3 Orygen, Parkville, Melbourne, VIC Australia; 4 Centre for Youth Mental Health, The University of Melbourne, Melbourne, Parkville, VIC Australia

**Keywords:** Virtual reality (VR), Psychosis, Informal carers, Communication skills, Feasibility study

## Abstract

**Background:**

Family (informal) carers play a vital role in providing care and support to relatives living with a psychotic disorder. Carers, however, often face challenges in communicating effectively with care recipients, particularly in the presence of psychosis symptoms such as delusions and hallucinations. Immersive technologies such as virtual reality have a growing evidence base in mental health treatments in patient groups, but their application to support carers has yet to be established. This study sought to evaluate the feasibility of CareVR, a therapist-facilitated virtual reality (VR) communication skills training programme for informal carers in psychosis. CareVR comprised four individual sessions designed to enhance carers' confidence and effectiveness in their conversations with a care recipient experiencing psychosis symptoms.

**Methods:**

An uncontrolled study design was conducted to ascertain the feasibility and acceptability of CareVR. The VR programme comprised two conversation scenarios between an adult avatar carer and an adult avatar relative (care recipient) with psychosis. Scenarios were delivered from both a third-person (observer) and a first-person (carer) perspective. In each scenario, participants selected how the avatar carer responded to communications from the avatar relative with psychosis. Following each training scenario, participants, supported by a facilitator, reflected on their experiences within the VR environment and their own lived experiences. They were also given opportunities to revisit and revise their choices. The final session focused on integrating learning across the training programme. Feasibility was assessed through recruitment, retention, session attendance, and completion of outcome measures. Acceptability was evaluated using participant feedback, including session satisfaction scores, and achievement of individualised goals.

**Results:**

The sample comprised twelve participants with diverse caregiving relationships and socio-demographic backgrounds. Recruitment targets were achieved, and all participants attended and completed the full training and assessment measures. Participants reported high levels of satisfaction for individual sessions, the intervention, overall, and goal achievement.

**Conclusion:**

Preliminary findings from our small and uncontrolled study suggest CareVR might be both feasible and acceptable to informal carers of individuals in psychosis. The study, however, was not designed to evaluate efficacy. Thus, any reports and conclusions of clinical benefits of CareVR require further investigation in a future adequately powered controlled trial.

**Clinical trial number:**

Not applicable.

## Background

People living with a psychotic disorder can experience a broad range of symptoms including distressing anomalous sensory experiences such as auditory verbal hallucinations (hearing voices) or unusual (delusional) beliefs about themselves, others, or events [1]. These symptoms can adversely affect the style and quality of their communications with and behaviours towards others [2, 3] including informal carers, who are typically relatives and close others providing unpaid care and support [4]. The United Kingdom (UK) has an estimated 6.5 million informal carers, many of whom support individuals with a psychotic disorder [5, 6]. Their support plays a vital role in promoting recovery and wellbeing, including improving treatment engagement and life expectancy, and reducing levels of relapse, hospitalisation and overall care needs and costs [7–9] However, key aspects of the caregiving role can be challenging, confusing, and adversely impact carers’ own wellbeing. For instance, carers may be exposed to difficult communications and behaviours from care recipients (e.g., persecutory delusions) as well as other problematic behaviours such as verbal aggression [10, 11]. These experiences can, in turn, adversely affect the style and quality of the caregiving relationship, and the effectiveness of carers’ communications and responses to care recipients [12, 13].

For some carers, negative communication styles may reflect the limited understanding of psychosis and its presentations. It might also reflect gaps in illness specific communication skills and confidence in establishing and maintaining positive interactions when care recipients experience delusions and hallucinations [14]. Caregiving relationships characterised by negative communication styles are robust predictors of poorer patient outcomes in psychosis, particularly elevated relapse rates and a need for more intensive care [15–17]. Further, negative caregiving relationships are associated with an increased risk of an overall breakdown in the caregiving relationship and care arrangements, and deterioration in patient outcomes [18] Thus, strategies to disrupt negative communication cycles in caregiving relationships and enhance carer management skills are important targets in treatment recommendations for carer interventions in psychosis [19–20]. However, we currently have a lack of evidence-based approaches to support carers in psychosis in developing, rehearsing, and implementing optimal communication styles with care recipients experiencing persistent delusions and/or hallucinations. Standard family-based interventions [21] are not designed to directly target carers’ cognitions, affect and communications in real time.

The last decade has seen growth and innovation in digital health technologies, including applications of virtual reality (VR) software in the assessment and treatment of mental health conditions [22, 23]. VR offers a unique opportunity to reproduce, control and manipulate ecologically valid environments and social interactions in real time through the use of avatars (virtual characters that individuals respond to as if they were social agents and in naturalistic settings) [24] Unlike role-play, which relies on imagination, VR provides a common reference point where the therapist can see exactly what the participant sees, facilitating more precise clinical formulation. VR also creates a “plausible” yet safe environment where nothing the individual fears can actually happen, allowing carers to freely experiment with and rehearse new communication styles, without the risk of real-world interpersonal consequences.

These approaches have been increasingly applied to support an individual’s learning and skill mastery to enable the transfer of skills in everyday life situations. VR assisted training has proven effective in improving understanding and reports empathy in carers of people living with dementia [25]. These technological developments have yet to be applied to carers in psychosis to address important and identified areas of need, of how best to communicate with the person they care for [26]. The current study sought to examine the feasibility and acceptability of CareVR, a prototype VR-assisted communication training package for informal carers of people with psychosis. Our two main research questions related to whether we could recruit, assess and retain participants in the VR-assisted training programme and is the VR-assisted training programme feasible to implement, acceptable to participants, and usable? Our secondary question concerned whether the training provided an indication of improvement in carer outcomes.

## Methods

The study received ethical approval from the King’s College London Research Ethics Board (reference number: HR/DP-22/23-34418). We have ensured the manuscript follows the RATE-VR guidelines [26] for reporting early-stage VR clinical evaluations, ensuring transparency in both the technical and clinical aspects of the study.

The uncontrolled study design incorporated quantitative and qualitative assessment methods.

### Recruitment and participants

Methodological literature, specifically Julious, [27] suggests a minimum of 12 participants per group as a “rule of thumb” for pilot trials to obtain reliable estimates for future power calculations. While larger pilot trials sometimes aim for *n* = 30 to better estimate feasibility parameters [28]. This study aimed to recruit a minimum of eight participants, which was considered sufficient for an uncontrolled study design to answer the primary research questions on basic feasibility, acceptability, and usability [29].

Participants were identified through public adverts via carer advocacy groups and social media. Prospective participants were screened by completing an online questionnaire or a telephone screen depending on their preference. Participants were eligible to take part if they self-identified as being in an informal caregiving role for an adult living with psychosis. For the study and consistent with previous studies and wider literature, carers were defined as family members, partners, close friends, providing care and support in an unpaid (informal) capacity and not within a professional, paid employed capacity [30]. Eligible participants could either live with the person with psychosis or live independently but maintain regular weekly contact. Participants had to be 18 years or older and reside in or close to the London area for logistical considerations.

People ineligible to participate were: (1) informal carers of adults with non-psychotic conditions; (2) people with medical conditions, which could be adversely impacted by VR use (i.e., epilepsy/seizures, active nausea, vomiting, and/or motion sickness [31]; 3) people with substantial hearing or visual impairments (i.e., blindness, severe hearing loss) precluding VR use [32, 33], or organic conditions (e.g., moderate dementia), to avoid confusion or disorientation [34] and 4) individuals unable to provide informed consent, or those needing an interpreter for comprehension.

### CareVR training protocol

#### The ‘getting ready’ session

After verifying eligibility, verbally consented participants attended a preparatory 60-minute getting ready session. During the session, participants were introduced to the VR equipment and scenarios. The session was designed to offer participants an opportunity to familiarise themselves with the equipment, its application and feel, and share any questions or concerns they might have had. Study consent forms and baseline measures were also completed during this session.

#### Training overview

Each 60-minute training session was scheduled for delivery over 4 consecutive weeks and held at King’s College London’s VR lab. The sessions, led by authors JO (Consultant Clinical Psychologist) and LR (Doctoral Trainee Clinical Psychologist), followed a structured protocol (i.e., participant exposure to VR scenarios + guided reflective discussion based on the participant’s experience in the VR and real-world carer experiences). The training package was developed from an iterative and collaborative process that embedded the lived experiences of people with psychosis and informal carers. Its therapeutic approach, inclusive of the therapist led discussion, and stance drew upon cognitive-behavioural framework, [35–37] cognitive behavioural therapy (CBT) evidence-based family interventions in psychosis, [21] carer focused interventions, [38] and research evidence on communication needs of families affected by psychosis and their impacts [39–41].

Consistent with ethical recommendations, [23]. CareVR was designed as a therapist-facilitated intervention, ensuring clinical safety and professional debriefing following VR exposure. Session 1, delivered from a third-person perspective, focused on observing the interaction and reflecting on the choices participants made, as well as the factors underpinning those decisions. Sessions 2 and 3, conducted from a first-person perspective, supported participants in making sense of the immersive experience, with particular attention paid to personal, illness-related, and situational factors influencing communication. Session 4 focused on consolidating key learnings and co-creating an individualised action plan to support the carer’s real-world wellbeing and communication needs.

#### VR equipment and session procedures

The training sessions used the Meta Quest 2 VR headset, which provided immersive 3D images and hand-tracking capabilities. VR headset adjustments were made for visual clarity and comfort, and sanitised before and after sessions. Participants experiencing any discomfort or unwanted effects could stop at any point and remove it.

CareVR scenarios were developed by the VR Lab at the Institute of Psychiatry, Psychology and Neuroscience, King’s College London. It was an iterative and collaborative process that embedded the lived experiences of people with psychosis and informal carers. This co-design approach was consistent with established best practices in digital interventions development, such as those described by Hardy et al. [42] and Adams et al. [43] which utilise lived experience and clinicians’ involvement to identify salient triggers and ensure clinical relevance. The specific scenario, a conversation between an avatar relative with psychosis (“Peter”) and his mother, was designed to simulate communication challenges typically observed within caregiving relationships where a care recipient experiences persistent delusions and hallucinations. CareVR uniquely utilises perspective shifts from an observer (third-person) to participant (first-person carer).

During exposure to VR scenarios, participants observed and interacted with avatars representing a person with psychosis (Peter) and how his verbal communications and behaviours towards his mother were affected by his psychosis symptoms. In the VR task, participants were presented with multiple-choice response options. Participants could select how Peter’s mother responds to Peter’s communications using the programme’s hand tracking features. These were designed to be as close as possible to responses a carer might make in real life, allowing for the assessment of “in the moment” communication styles. Both VR scenarios lasted approximately three minutes.

#### Training session 1

**Fig. 1:**
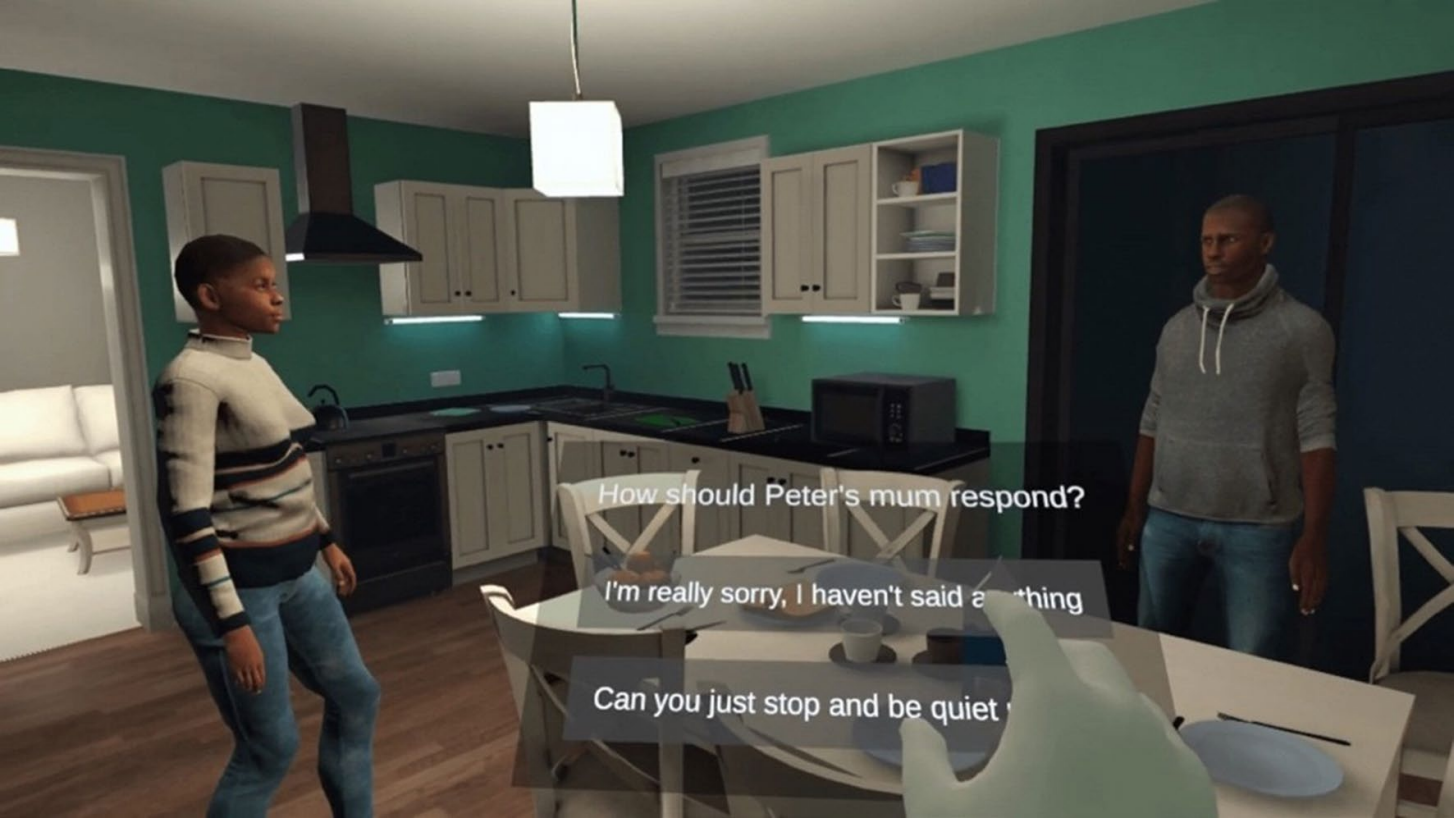
Peter (right) and his mother (left) during the first scenario, which was presented from a third-person perspective. Participants would observe/listen to the conversation, selecting responses to determine how the mother responds

Participants observed a simulated verbal and behavioural interaction between Peter and his mother (see Fig. 1) from a third-person (observer) perspective. The interactions between Peter and his mother were directly impacted by Peter’s psychosis symptoms. During this session, participants were asked to select the carer response options that were as close to a response they would make. Their choices and the factors underpinning their decisions and overall observations were important material for the guided reflective discussions that followed.

#### Training sessions 2–3

**Fig. 2:**
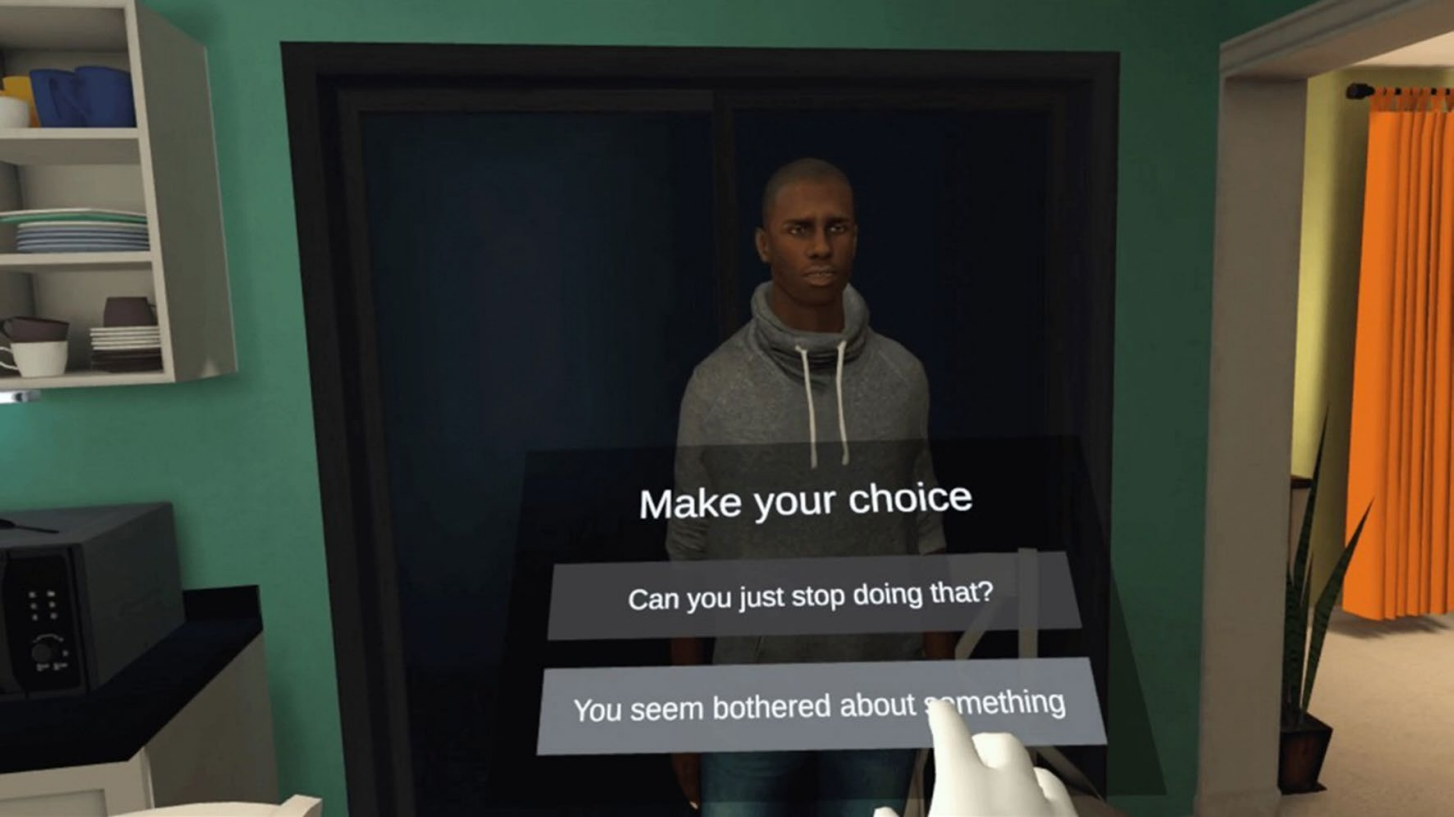
Example of Scenario 2 and response choices

Across sessions two and three, participants were re-exposed to Peter’s verbal and behavioural communications from the previous session, but this time they experienced it from the first-person perspective of the carer avatar, as if they were engaging directly with Peter (Fig. 2). The perspective shift from observer to first person was designed to facilitate and expedite the first-hand experience of the carer. Peter’s presentation range and response options remained identical to what was shown in training session 1. Guided reflective discussions facilitated participants’ sense making of their immersive experiences and understanding of the pertinent factors (e.g., personal, illness-related, situational, relationship) that can impact their communication styles.

#### Training session 4

The 4th and final training session consisted of a facilitated discussion between the participant and therapist to reflect on and integrate key learnings from the training sessions for their carer experiences and wellbeing, and an individualised action plan moving forward.

### Post-training and follow-up

Participants completed a 60-minute post-training assessment session, with follow-up data collection scheduled one month later. The post-training and follow-up sessions comprised the same measures completed at baseline.

### Measures

Assessment measures were collected at three time points: pre-training (baseline), post-training, and one-month follow-up. Questionnaires were administered in a specific order to minimise mood-related influence on caregiving appraisals. Participants were reimbursed (£15 per hour) for their time and input for each data collection session.

Socio-demographic data were collected comprising participant’s age, gender, ethnicity, employment status, relationship status, living arrangements, and estimated hours per week they were engaged in caregiving activities. Participants completed a single item and open-ended question exploring common types of communication difficulties they experienced in their caregiving relationship (i.e., can you describe the most common types of communication difficulties, if any, that you experience with the person with psychosis you support?).

#### Primary outcome measures

Data were collected to explore the extent to which the study was able to recruit and retain participants in the training, in accordance with the protocol. Data included information around initial interest in participation, numbers screened, reasons for exclusion, and rates of informed consent, session attendance, training drop-out rates, and outcome measure completion. Participant feedback was also collected after each session. This feedback focused on recording their level of satisfaction with each session, rated from 1 (not satisfied at all) to 10 (most satisfied) and beliefs about aspects of the session that were considered the least and most helpful.

Goal Attainment Scaling (GAS) [44] was used to provide individualised assessments of training outcomes, as it is highly sensitive to detecting personalised accomplishments that standardised scales may miss. It has been previously used in VR studies with good content and construct validity [45, 46].

The Presence Questionnaire, [47] a frequently used self-report measure of presence, was used to capture a participant’s perception of presence and sense of being within a simulated environment. Participants completed four of the six applicable questions, with the higher yielding scores indicating the greatest sense of presence. To measure whether participants experienced any adverse side effects during the VR training, an adapted version of the Oxford Virtual Reality Side Effects Checklist (O-VRSE) was used [48]. The O-VRSE explored the potential physical and psychological effects of wearing a VR headset; the psychological experience of the simulations; and the potential physical and psychological effects after being in VR. These included feelings of sickness, difficulties distinguishing between the participant’s real and virtual worlds, and potential positive experiences. Nine non-applicable questions were removed from the finalised questionnaire. Examples of items removed included: “wearing the headset made my voices worse for the rest of the day.” Participants commenced each session with completion of the Clinical Outcomes in Routine Evaluation (CORE-10) questionnaire to monitor weekly levels of distress [49].

### Data analyses

Descriptive analyses summarised data on feasibility and acceptability markers, including rates of participant recruitment and retention. Data from participant feedback about the training were analysed informed by principles of thematic analysis [50]. Key themes were identified from written feedback and presented alongside illustrative quotations.

## Results

### Feasibility

**Fig. 3:**
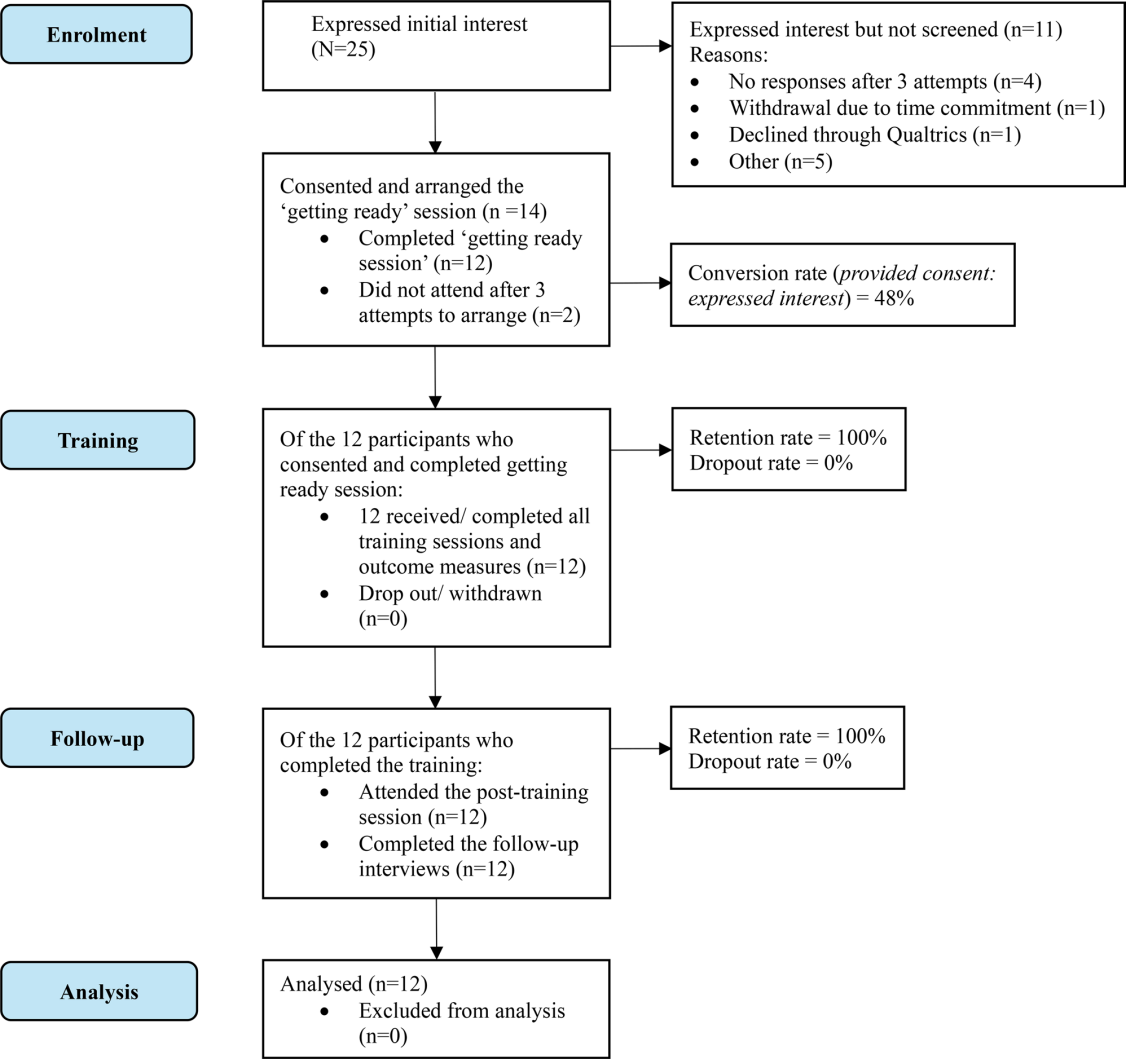
Recruitment flowchart

Participants were recruited in a six-month window (August- December 2023) with approximately half recruited through local carer support and advocacy groups. Twenty-five participants expressed initial interest in the study. Thirteen were not screened or decided not to continue due to the time commitment required, exclusionary health difficulties (e.g., motion sickness and epilepsy), and loss of contact with the researcher. Twelve of the 25 carers who expressed initial interest in participating, provided consent to participate, which reflected a 48% conversion rate. All 12 participants completed the training, including their baseline, post-training, and follow-up outcome measures (100%). There were no dropouts or missing data to report. The recruitment process is summarised in Fig. 3 below.

### Sample characteristics

The sample had a mean age in their late 50s, and around two-thirds of participants were female, and most were in paid employment. More than half lived with the care recipient and provided an estimated 30 h or greater of caregiving per week. Participants were mostly in caregiving roles for their adult male children. See Table 1 for the full breakdown of participant demographics and characteristics.

**Table 1:** Participant demographics and characteristics

Characteristic	Frequency (%)
**Age**	
36–45	1(8.3%)
46–55	2 (16.7%)
56–65	7 (58.3%)
66–77	2 (16.7%)
Mean (SD)	59.6 years (9.62)
**Gender**	
Female	8 (66.7%)
Male	4 (33.3%)
**Ethnicity**	
Asian or Asian British	1 (8.3%)
Black, Black British, Caribbean, or African	3 (25%)
White British	5 (41.7%)
White Other	1 (8.3)
Other ethnic group	2 (16.7%)
**Partnership Status**	
Cohabiting (living with partner)	2 (16.7%)
Married or registered in civil partnership	4 (33.3%)
Not currently in partnership (single)	4 (33.3%)
Separated, but still legally married or still legally in civil partnership	1 (8.3%)
Widowed or surviving civil partnership partner	1 (8.3%)
**Current employment status**	
Employed full-time	6 (50%)
Employed part-time	1 (8.3%)
Other	2 (16.7%)
Retired	2 (16.7%)
Voluntary employment	1 (8.3%)
**Care recipient is**	
Mother	2 (16.7%)
Daughter	1 (8.3%)
Son	6 (50%)
Partner	1 (8.3%)
Other	2 (16.7%)
**Living with the person cared for**	
Yes	7 (58.3%)
No	5 (41.7%)
**Hours of weekly contact with the person cared for**	
5 h or less	1 (8.3%)
Between 5–10 h	2 (16.7%)
Between 10–15 h	1 (8.3%)
Between 15–20 h	1 (8.3%)
More than 30 h	7 (58.3%)
**Other caring responsibilities**	
Yes	2 (16.7%)
No	10 (83.3%)
**Length of time providing care for the person (years)**	
1–10	5 (41.7%)
11–20	3 (25%)
21–30	2 (16.7%)
31–40	2 (16.7%)
**Someone to confide in**	
Yes	9 (75%)
No	3 (25%)

**Table 2:** Participant feedback on training: key themes

Theme	Illustrative quotes
**Deeper personal reflection**	“Really thinking about what is going on with me when engaging with my son.”
	“First person experience helped me experience empathy for the mother, and my own thoughts for how I might react, all without judgement.”
	“Being able to self-evaluate me in certain situations where I wouldn’t normally think about to the level of detail.”
**Emotion recognition and validation**	“Understanding that my feelings are helpful” … “I feel really happy with talking about how I feel”
	“Acknowledging that caring comes with feeling fear. It was good for me to discuss the emotions that I felt today”
	“I learned I judge myself too harshly. This week I found it helpful in that I am unaware that I am possibly keeping my feelings unchecked”
**Understanding communication impacts**	“Understanding how negative responses are not helpful”
	“Exploring different options in communication, tone. Focusing on what’s going on for me. Discussion with [facilitators]”
	. “Discussing possible consequences and risks that come with caregiving”
**Thinking about own needs**	“Understanding that I don’t always put myself first.”
	“Learning more about how I can help my son by looking after myself.”
**Feeling listened to**	Being able to discuss my feelings to persons who understands” … “I am glad I can open up issues that bothers me” … “I wish more professionals took time to really listen to clients and relatives by remembering their cultures and experiences.”
	“I was listened to and given support with my feelings and circumstances.”

### Communication difficulties

Participants described a range of communication difficulties they often encountered in their carer role. These commonly included: circumstances where psychosis symptoms left carers somewhat fearful of saying the wrong thing and making things worse (e.g., ‘he can become suspicious. I am conscious that things might annoy him’) along with difficulties in a care recipient’s expressive communication (e.g., ‘It can be difficult at times to follow along to the conversation’) and emotional regulation (e.g., ‘There are 5 moods that influences the situation. Anger at me is particularly hard to deal with and his despair’).

### Acceptability

75% of participants rescheduled at least one session due to their own scheduling constraints.

Participants reported high levels of satisfaction with the Getting Ready sessions, with average ratings of 9 out of 10. They valued the opportunity to familiarise themselves with the VR equipment and clarify any queries and areas of uncertainty about the training process and their involvement. Satisfaction with the VR training sessions was also high, with average ratings of 9 out of 10 across all four sessions.

The VR scenarios were perceived as being useful in supporting self-reflection, emotional validation, and enhancing understanding of carer experiences. Lower satisfaction was associated with participant’s desire for longer sessions (i.e., sessions to extend beyond 60 min), expanded VR capabilities and adjustments to emotional intensity of the training scenario. See Table 2 for the key themes identified from participant feedback about the training and illustrative quotes.

### Outcome measurement

All participants completed every outcome measure at each of the three-time intervals (100% completion rate). Baseline assessments took approximately 37 min on average, while post-training and follow-up assessments averaged around 47 min.

#### Goal attainment scaling

At baseline, all participants identified at least two training goals. Each participant set goals focused on learning strategies to enhance communication, coping skills, self-care, and wellbeing. Post-training, most participants reported substantial progress towards their goals. This was reflected by the most frequent ratings on the GAS question being “much more than expected” (50%) and “more than expected” (33.3%). These gains were maintained at follow-up, with participants mostly commonly endorsing “more than expected” (50%) and “much more than expected” (25%).

#### VR sense of presence, positive and negative VR experiences

Participants reported a moderate-to-high sense of presence in the VR scenarios, with 65% endorsing the highest rating for feeling immersed in the virtual environment. All participants endorsed positive experiences with VR including feelings of comfort, optimistic, and excitement. Negative experiences were infrequent (*n* = 8.3% of sessions) and largely involved confusion or minor physical discomfort related to the VR headset.

## Discussion

This study sought to investigate the feasibility of implementation and the acceptability of CareVR, a therapist-led, VR-based communication skills training programme specifically designed for carers of people with psychosis. Twelve participants consented to participate, which exceeded the initial recruitment target of eight participants. Further, all twelve carers completed the full training programme and assessments, which offered support for retention outcomes. The encouraging retention rate aligns with previous research exploring the feasibility of psychological interventions for carers of people with psychosis [51, 52]. Our findings suggested that once carers provided consent to participate, they were more likely to remain engaged with the process, which might offer further support for the practical feasibility of the VR training. The retention rate might also be explained by the complementary cognitive and behavioural components of the training programme, which offered timely feedback to participants on their learning. It is equally possible that retention rates reflect the benefits of the brevity of the overall training, which might increase the likelihood of participants feeling able to fit the training in their already busy schedules. Carers are often time poor [53] and can miss out on different development opportunities, including those that can advance their wellbeing when they do not have time [54]. The merit of having a brief targeted intervention is consistent with reviews and other feasibility studies applying brief interventions for carers of people with psychosis [24, 55]. Offering brief interventions, like the VR training, may help to effectively engage carers whilst potentially being more feasible to implement.

The study was able to recruit carers with a diversity in socio-demographic characteristics, including age, gender, race and ethnicity, and types of caregiving relationships. That said, the sample yielded a much larger proportion of female parental carers. The higher proportion of participants who were the mothers of care recipients is consistent with patterns commonly observed within the wider literature for carers of people with psychosis [56]. Further, the study sample comprised almost two-thirds from ethnically minoritised backgrounds. Informal care is not a “one size fits all” experience and participants are likely to start training with varying relational dynamics, needs, difficulties, and ways of coping. Although different carer groups (e.g., siblings, partners, mothers, fathers, children) can experience similar issues (e.g., emotional distress, concerns for the future), the experiences of one type of carer (mothers, minoritised ethnic groups) cannot be assumed to generalise to other groups who might differ in several ways including how they cope with illness impacts and make sense of the illness [57] Ensuring the inclusion of a broader range of carer groups in future studies would be beneficial.

Levels of participant completion of outcome measures were good. Participant measures were completed across the getting ready, training, post-training, and follow-up sessions. The high completion rates are likely to reflect, in part, the in-person administration of measures. Though assessment measures were seemingly acceptable, participant feedback suggests the need for potential modifications in their administration, which included for one participant, the opportunity to complete assessments on a desktop computer instead of an iPad. Ensuring participants are not unnecessarily deterred from engagement and completion, due to the study methods, will be an important consideration going forward [58].

The training appeared to be acceptable to participants, as reflected by their weekly satisfaction scores. Participants’ self-reported average scores of nine out of ten across all sessions indicated strong levels of satisfaction and positive training experiences. High levels of acceptability were also evidenced by the descriptive analysis of participants’ individualised goals on the goal attainment scaling (GAS). In this study, 50% of participants achieved “much more than expected” progress post-training. For a future randomised controlled trial (RCT), GAS would remain a strong candidate for a primary outcome, as it has been successfully used as the primary measure in other VR-assisted trials (e.g., V-NeST)[59] to reflect personal recovery goals. Alternatively, a validated measure of communication confidence or caregiving impact, areas where significant improvement was indicated qualitatively, could serve as a primary clinical endpoint.

Post-training GAS scores offered positive signals about progression, which might suggest that participants may have been able to learn helpful coping and communication strategies. Similarly, albeit proposed somewhat cautiously, the follow-up GAS ratings might also signal potential sustained positive effects of the training, as these were similar to post-training scores. However, one participant described their difficulty applying the learning outside of the training sessions. This may reflect the emotional stress associated with their role, and the unpredictable nature of psychosis, which can make it harder for some carers to implement the learning in everyday situations [60].

Participant feedback further corroborated the training’s acceptability and potential impact. Participants highlighted that learning the importance of prioritising their own needs was one of the most helpful experiences. This was an important knowledge aspect of the training, as carers who prioritise their own physical, emotional, psychological, and spiritual health report feeling better equipped to manage the challenges associated with informal care in psychosis [61]. Approximately half of the participants described how the VR helped facilitate meaningful discussions around their personal caregiving experiences.

It is important to remain cognizant of areas noted by participants as being least helpful or suitable for improvement (e.g., a preference for longer training sessions, more scenarios and avatar responses). It could be argued that these would be conceivable areas to focus attention, going forward, having established the initial feasibility. It is also possible that if carers have had a good and positive training experience that included a space where they felt safe to share some of the innermost concerns about their caregiving and felt listened to, then a request for longer training sessions and avatar scenarios and dialogue would not be unexpected. Future investigations that can disentangle the active contribution of the training to carer outcomes versus the therapeutic effects of providing a safe listening environment are needed as part of the development of this work.

## Limitations

This was an uncontrolled study with a small sample size. Thus, it was delivered in the absence of control groups, randomisation, and assessor blinding. Nevertheless, as a study focused on exploring feasibility and acceptability, the inclusion of such methodological features would not have been appropriate at this preliminary stage. The use of open recruitment may have introduced selection bias, potentially attracting participants with greater motivation to engage in research. In addition, feasibility and accessibility outcomes may have benefited from more robust operationalisation to reduce the risk of researcher bias and subjectivity. For example, applying pre-defined measure, such as the Avery and colleagues’ traffic light system could have enabled a more structured and transparent review of study processes [62]. This approach could have provided a more standardised method for evaluating the primary aims of the study.

A further limitation relates to the use of convenience sampling, which might have increased the risk of selection bias. Most participants were recruited through carer support groups, which may have limited the representativeness of the sample.

Although these groups comprise a diverse range of carers, they may not reflect the experiences of individuals without access to support, for whom discussing personal experiences may feel unfamiliar or uncomfortable. Any future scale- up of the training should carefully determine an appropriate sample size and implement strategies to enhance representatives.

No formal assessment of intervention fidelity was undertaken. Adherence to and consistency with the intervention protocol were monitored informally through weekly post-session debriefs. However, as emphasised by quality assessment tools, it is important to incorporate suitable methods to measure the consistency of an intervention’s delivery [63]. Given that fidelity has been shown to be an important outcome mediator [64, 65] assessing for fidelity in future replications may help increase the reliability and validity of any findings.

## Conclusion

As part of an uncontrolled design with a self-selected volunteer sample, this study reports the feasibility and acceptability phase of complex intervention development, consistent with the framework outlined by Skivington et al. [66] While the current results cannot speak to clinical benefits, the preliminary findings offer encouraging signals that CareVR, a novel, therapist-led VR communication skills training for carers of people with psychosis, can be successfully delivered and evaluated within this context, demonstrating potential acceptability among carers.

Building on these findings, several next steps are proposed. First, the intervention will undergo iterative refinement, incorporating participant feedback to enhance interactivity and appropriately calibrate the emotional intensity of scenarios. Second, a standardised treatment manual will be developed to support intervention fidelity and reproducibility in subsequent trials, in line with recommendations for complex interventions [66]. Third, a pilot randomised controlled trial will be conducted to provide preliminary evidence of efficacy while further assessing feasibility in a more rigorous study design [67]. Finally, implementation will be systematically evaluated using established frameworks such as the NASSS framework [68] to examine how CareVR can be integrated into existing clinical workflows and service systems.

Despite these considerations and potential need for refinement, CareVR shows promise as an intervention that could address the needs of an underserved group in mental health care.

## Data Availability

All data generated or analysed during this study are included in this published article.

## Abbreviations

VR: Virtual Reality
CBT: Cognitive Behavioural Therapy
O-VRSE: Oxford Virtual Reality Side Effects Checklist
CORE-10: Clinical Outcomes in Routine Evaluation
GAS: Goal Attainment Scaling
